# Paediatric preoperative sedation practices in tertiary maternity and children’s hospitals in China: a questionnaire survey

**DOI:** 10.1186/s12887-021-02802-0

**Published:** 2021-08-09

**Authors:** Bo Li, Huiyan Hou, Jie Bai, Mazhong Zhang, Shengde Li, Jijian Zheng

**Affiliations:** 1 Department of Anaesthesiology, Shanghai Children’s Medical Center, National Children’s Medical Center, 1678 Dongfang Road, Shanghai, China; 2 Paediatric Clinical Pharmacology Laboratory, Shanghai Children’s Medical Center, National Children’s Medical Center, 1678 Dongfang Road, Shanghai, China; 3 Department of Anaesthesiology, Qingdao Women and Children’s Hospital, 217 Liaoyang Xi Road, 266000 Qingdao, Shandong China

**Keywords:** Child, China, Preoperative sedation, Questionnaires, Surveys

## Abstract

**Background:**

Preoperative anxiety is a common problem in the paediatric population, and several studies have reported that it is related to adverse events such as emergence delirium and postoperative psychological and behavioural changes. In recent years, increasing attention has been paid to paediatric preoperative anxiety in China. A variety of strategies, including sedatives, parental presence, and audio-visual interventions, have been used to relieve paediatric preoperative anxiety, but there is no well-recognised procedure for paediatric preoperative sedation. Therefore, this study aimed to investigate current paediatric preoperative sedation practices in tertiary children’s hospitals in China.

**Methods:**

All tertiary maternity and children’s hospitals registered with the National Health Commission of the People’s Republic of China were invited to participate in an electronic survey, which included information on the preoperative sedation caseload, sites where preoperative sedation was performed, preoperative sedation methods used in different age groups, choice of sedatives, contraindications for premedication, staff structure for sedative administration and monitoring, and patient-monitoring practices.

**Results:**

All 81 hospitals participating in our study completed the survey, and 38 hospitals (46.9 %) provided their preoperative sedation protocols. Twenty-four hospitals performed fewer than 5,000 preoperative sedation cases annually, and 9 hospitals performed more than 10,000 cases annually. Preoperative sedation was performed in preoperative preparation areas, preoperative holding areas, and operation rooms in 47.4 %, 26.3 %, and 13.2 % of hospitals, respectively. Sedatives were the most used interventions for paediatric preoperative sedation in all age groups, and the most widely used sedatives were propofol (intravenous) and dexmedetomidine (intranasal). The most common contraindications were American Society of Anesthesiologists class ≥ 3, emergency operation, and airway infection within 2 weeks. Sedatives were administered mainly by anaesthesiologists (63.2 %), and children were monitored mainly by anaesthesiologists (44.7 %) and nurses (39.5 %) after administration. Pulse oximetry was the most widely used monitoring device.

**Conclusions:**

Fewer than half of the tertiary maternity and children’s hospitals in China provide paediatric preoperative sedation service, and the service practices vary widely. Further improvements are required to ensure the quality of paediatric preoperative sedation services and establish standard operating procedures.

## Background

Preoperative anxiety is a subjective feeling of tension and apprehension in patients before surgery. It is very common in preoperative patients, especially paediatric patients, which is reported that 65 % paediatric patients suffer from preoperative anxiety [1]. Many factors, including parental separation, fear of physicians, unfamiliar environments, and needle phobia can induce preoperative anxiety. Serious preoperative anxiety is thought to be related to emergence delirium, postoperative psychological and behavioural changes, and adverse events [2, 3]. A variety of strategies, including sedatives, parental presence, audio-visual interventions and transporting children in toy cars, have been used to relieve paediatric preoperative anxiety [4–6], but there is no well-recognised procedure or guidelines for paediatric preoperative sedation so far. Although American Society of Anesthesiologists (ASA) guidelines for moderate procedural sedation can provide a certain reference for paediatric preoperative sedation, especially in patient evaluation and monitoring [7], but it might not be appropriate to use ASA procedural sedation guideline to direct paediatric preoperative sedation practice. Furthermore, different races, cultures, education levels, income, etc. factors also affect the incidence and alleviating strategies of preoperative anxiety [8, 9]. Therefore, we aimed to investigate current paediatric preoperative sedation practices in tertiary maternity and children’s hospitals, in the hope of contributing to the future establishment of a standardised practice of paediatric preoperative sedation in China and provide reference for other countries.

## Methods

**Fig. 1:**
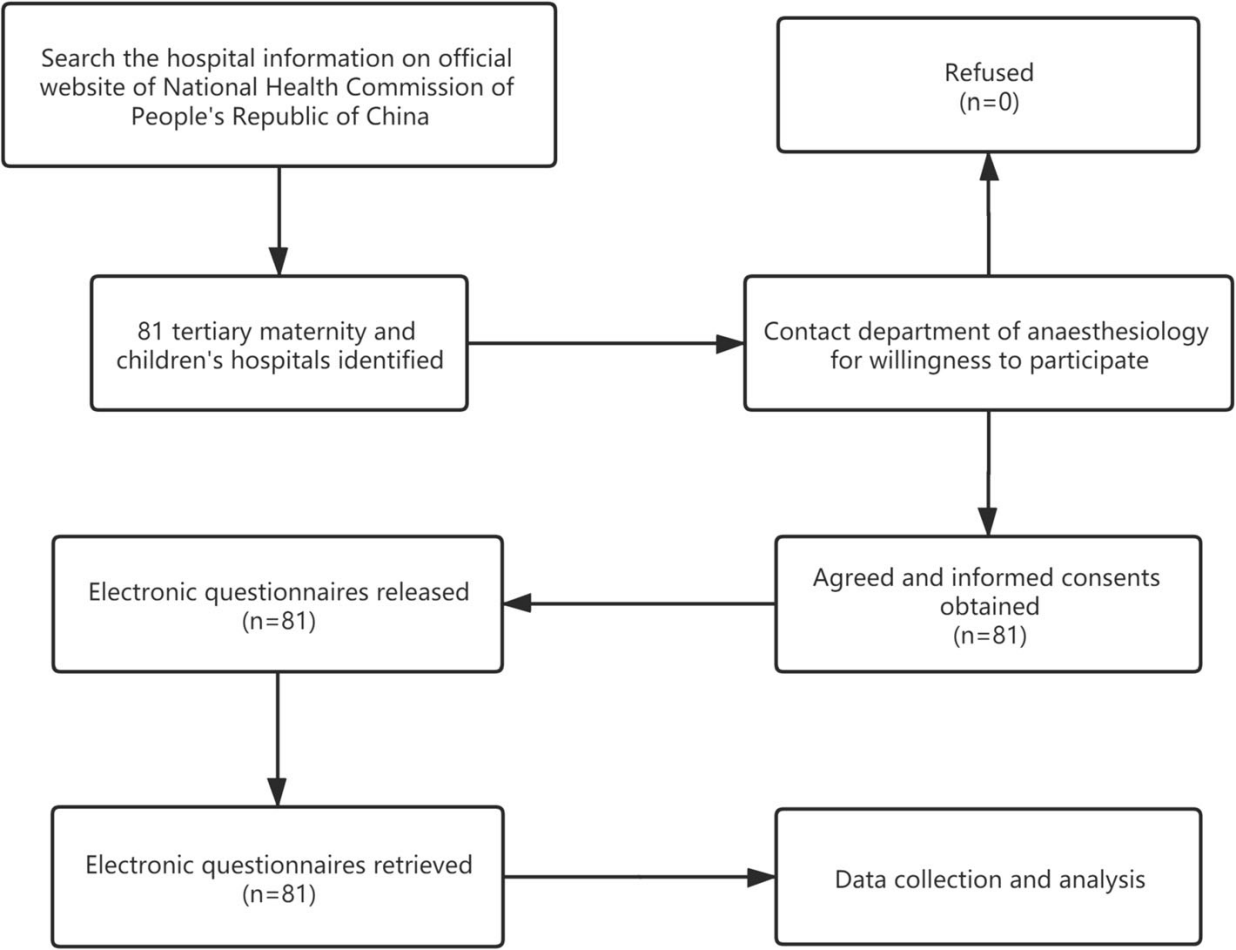
The flow diagram of the study

This study was reviewed by the Institutional Review Board of Shanghai Children’s Medical Centre. As the study did not involve access to private patient data or alterations of paediatric preoperative sedation practices, it was exempted from the need for ethical approval and was deemed approved. We first identified all the tertiary maternity and children’s hospitals based on the hospital register information listed on the official website of the National Health Commission of People’s Republic of China and then contacted the department of anaesthesiology using the registered hospital contact information for willingness to participate in this survey. After informed consent was obtained, we built a WeChat Survey Group using a smartphone and generated an electronic questionnaire through the in-built WeChat Mini Programme. To avoid more than one reply from the same hospital, only one anaesthesiologist from each hospital was invited to join this WeChat Survey Group. The questionnaires were released in May 2020. The participants of this study were asked to report official statistics of their hospitals whenever possible, and they were allowed at least 1 month for data collection. The questions in the survey were related to the preoperative sedation caseload, sites where preoperative sedation was performed, preoperative sedation methods used in different age groups, choice of sedatives, contraindications for premedication, staff structure for sedative administration and monitoring, and patient-monitoring practices. The flow diagram of the study is shown in Fig. 1.

## Results

Eighty-one tertiary maternity and children’s hospitals were invited to join the study, and electronic questionnaires were sent to representatives from each of the 81 hospitals. All hospitals completed the questionnaires. Of the 81 hospitals, 38 (46.9 %) provided preoperative sedation services. At least one hospital in each province and municipality in China was included, except for the Tibet Autonomous Region and the Ningxia Hui Autonomous Region. According to the National Health Commission of People’s Republic of China, there are no tertiary maternity and children’s hospitals registered in these two regions.

### Preoperative sedation caseload

**Fig. 2:**
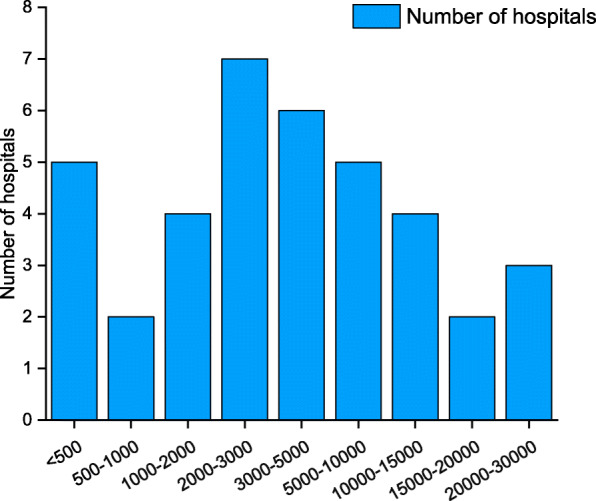
Caseload of paediatric preoperative sedation in tertiary maternity and children’s hospitals in China

Of the 38 hospitals that provided paediatric preoperative sedation, 24 hospitals performed fewer than 5,000 cases of preoperative sedation per year. Five hospitals performed 5,000–10,000 cases annually, and 9 hospitals performed more than 10,000 cases annually. Details are shown in Fig. 2.

### Sites where preoperative sedation was performed

**Fig. 3:**
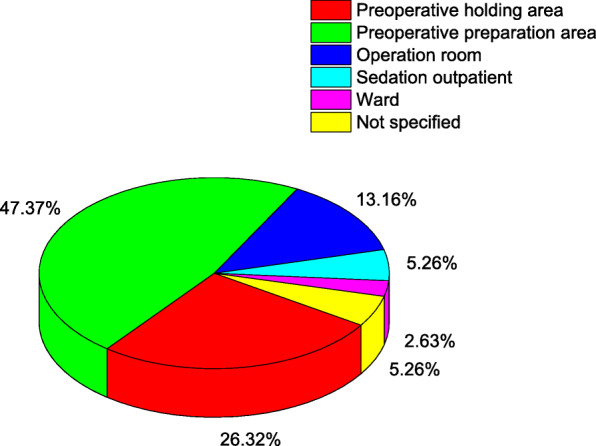
Sites where preoperative sedation was performed in tertiary maternity and children’s hospitals in China

Paediatric preoperative sedation was performed mainly in the preoperative preparation area (47.4 %), preoperative holding area (26.3 %), and operation room (13.2 %). A minority of the paediatric preoperative sedation cases were performed in outpatient clinics with facilities for sedation and wards. The details are shown in Fig. 3.

### Preoperative sedation methods used in different age groups

Methods of paediatric preoperative sedation varied in our study. Premedication was the most common method for paediatric preoperative sedation in all age groups (> 80 %). Nipple sucking was another alternative for children under 3 years of age, and the usage of nipple sucking decreased with increasing age. Audio-visual interventions, including cartoons, video games, virtual reality glasses, and background music, were more commonly used for children aged 1–3 years (43.2 %) and 4–6 years old (51.7 %). Distractions including clown doctors, toys, picture stories, and comic books, were more popular among infants (52.4 %) and children aged 1–3 years (62.2 %) and 4–6 years (58.6 %). Surprisingly, parental presence was not the first choice for any age group. Details are shown in Table 1.

**Table 1:** Current practices of preoperative sedation in children of all age groups in tertiary maternity and children’s hospitals in China

Age group	Method chosen for preoperative sedation	Number of hospitals (*n*=)	Proportion (%)
Newborn (*n* = 6)	Sedatives	5	83.3
	Nipple sucking	5	83.3
	Parental presence	2	33.3
	Audiovisual intervention	2	33.3
	Distraction	2	33.3
< 1 year old (*n* = 21)	Sedatives	17	81.0
	Nipple sucking	7	33.3
	Parental presence	10	47.6
	Audiovisual intervention	5	23.8
	Distraction	11	52.4
1–3 years old (*n* = 37)	Sedatives	36	97.3
	Nipple sucking	8	21.6
	Parental presence	21	56.8
	Audiovisual intervention	16	43.2
	Distraction	23	62.2
4–6 years old (*n* = 29)	Sedatives	28	96.6
	Parental presence	15	51.7
	Audiovisual intervention	15	51.7
	Distraction	17	58.6
> 7 years old (*n* = 14)	Sedatives	12	85.7
	Parental presence	5	35.7
	Audiovisual intervention	5	35.7
	Distraction	5	35.7

### Choice of sedatives

**Fig. 4:**
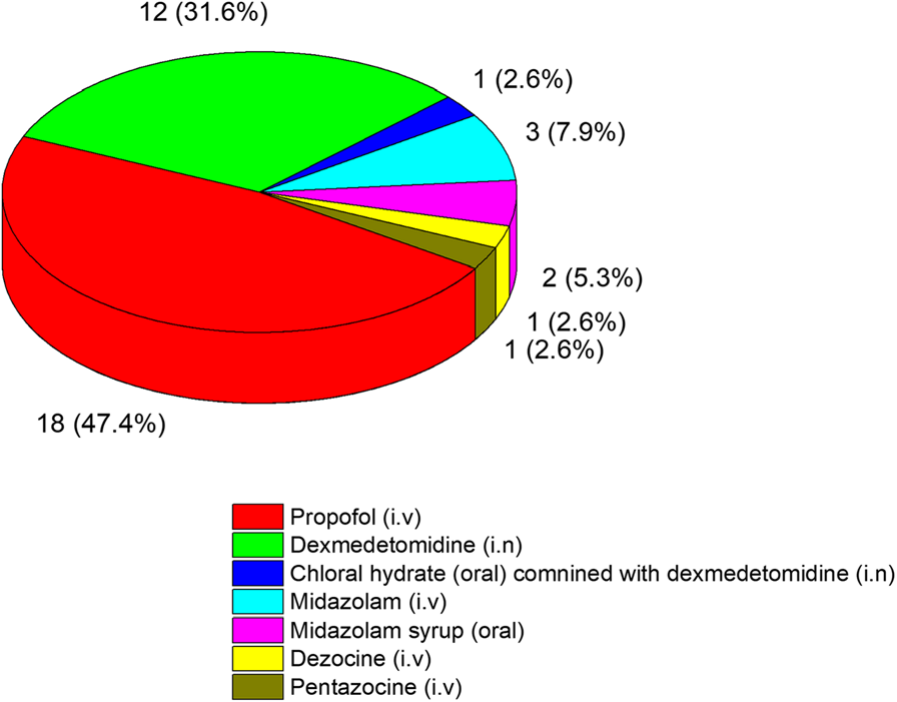
Choice of sedatives for paediatric preoperative sedation in tertiary maternity and children’s hospitals in China

The most common sedatives used for paediatric premedication were intravenous propofol (47.4 %), intranasal dexmedetomidine (31.6 %), and intravenous midazolam (7.9 %). In addition, oral midazolam and intravenous dezocine and pentazocine were also reported. Combined administration of oral chloral hydrate and intranasal dexmedetomidine was reported in one hospital. The details are shown in Fig. 4.

### Contraindications for premedication

**Fig. 5:**
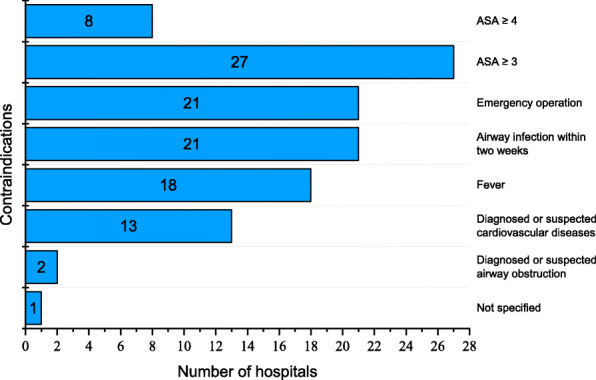
Contraindications for paediatric premedication in tertiary maternity and children’s hospitals in China

Several complications were reported as contraindications for paediatric premedication in our study, including ASA classification ≥ 3 or ≥ 4, emergency operation, airway infection within 2 weeks, fever, diagnosed or suspected cardiovascular diseases, and diagnosed or suspected airway obstruction. Of the contraindications reported, ASA classification ≥ 3 (71.1 %), emergency operation (55.3 %), and airway infection within 2 weeks (55.3 %) were the most common. Details are shown in Fig. 5.

### Staff structure for sedative administration and monitoring

Sedatives were administered by anaesthesiologists in 24 (63.2 %) hospitals and nurses in 10 (26.3 %) hospitals. After administration, patients were monitored by anaesthesiologists in 17 (44.7 %) hospitals and nurses in 15 (39.5 %) hospitals. Four hospitals did not specify the sedative administration personnel and six hospitals did not specify the monitoring personnel (Table 2).

**Table 2:** Staff for sedative administration and monitoring of preoperative sedation in tertiary children’s hospitals in China

Staff structure	Sedative administration (*n*=)	Monitoring (*n*=)
Anaesthesiologists	24 (63.2 %)	17 (44.7 %)
Nurses	10 (26.3 %)	15 (39.5 %)
Not specified	4 (10.5 %)	6 (15.8 %

### Patient-monitoring practices

Pulse oximetry was the most commonly used monitoring device (29 hospitals, 76.3 %). Respiratory rate, electrocardiography, and non-invasive blood pressure were used in 22 (57.9 %), 14 (36.8 %), and 11 (28.9 %) hospitals, respectively (Table 3).

**Table 3:** Monitoring events used in paediatric preoperative sedation in tertiary children’s hospitals in China

Monitoring devices	Number of hospitals (*n*=)	Proportion (%)
Pulse oximetry	29	76.3
Respiratory rate	22	57.9
Electrocardiography	14	36.8
Noninvasive blood pressure	11	28.9
Not specified	8	21.1

Continuous and intermittent patient-monitoring were reported in 16 and 9 hospitals, respectively, while 13 hospitals did not specify the monitoring practice. Details are shown in Table 4.

**Table 4:** Modes of monitoring used in preoperative sedation in tertiary children’s hospitals in China

Monitoring modes	Number of hospitals (*n*=)	Proportion (%)
Continuous	16	42.1
Intermittent		
every 5 min	6	15.8
every 10 min	2	5.3
every 15 min	1	2.6
Not specified	13	34.2

## Discussion

Our results demonstrate that less than half the tertiary maternity and children’s hospitals in China provide paediatric preoperative sedation and pharmacological interventions, including intravenous propofol and intranasal dexmedetomidine, are preferred over non-pharmacological interventions. Although intravenous administration of propofol is the first choice of paediatric preoperative sedation, intranasal dexmedetomidine is gaining popularity. Preoperative preparation areas are the most commonly used areas for sedative administration. Sedative administration and patient monitoring during paediatric preoperative sedation is performed mainly by anaesthesiologists. Pulse oximetry and respiratory rate is monitored in most hospitals during preoperative sedation.

Although the importance of paediatric preoperative sedation has been repeatedly emphasized recently, only 46.9 % of tertiary maternity and children’s hospitals in China provide preoperative sedation; furthermore, over 47 % sedation was performed in preoperative preparation areas without considering the waiting and separating anxiety in preoperative holding areas. A shortage of clinical anaesthesiologists and anaesthesia nurses, unreasonable structural layout of operating room, sedative availability, and lack of preoperative sedation knowledge and training might be the possible reasons that paediatric preoperative sedation services were not performed well in China. Great efforts have been made to increase the number of anaesthesiologists by National Health Commission of People’s Republic of China; however, it might take some time to overcome this issue since physician anaesthesia providers are fewer than 6 per 100,000 population in China, while this number is 20.82 in United States and 18.60 in European [10].

Differing from oral midazolam as the mainstream paediatric preoperative sedation in the United States and our hospital, 47.4 % of tertiary maternity and children’s hospitals in China used intravenous propofol as the first choice of preoperative sedation [11]. Vein opening in ward and fast paced turnover of surgical patients might be the reasons for using intravenous propofol as the first choice of preoperative sedation in China. With the recent introduction of dexmedetomidine in China, intranasal dexmedetomidine sedation has become popular and shows a satisfactory effect on children’s behaviour on separation from parents [12]. With increasingly promising evidence regarding the efficiency and safety in Chinese children, dexmedetomidine is expected to replace propofol as the most widely used sedative in paediatric premedication in China in the future [13, 14].

In contrast to pharmacological interventions, non-pharmacological interventions were used less in tertiary maternity and children’s hospitals in China. Many hospitals reported using combined non-pharmacological interventions (e.g., background music, cartoons, and toys) to supplement pharmacological interventions in our study. To our surprise, parental presence was not the first choice of non-pharmacological interventions in any of the age groups in our study. The reason for this may be that parental presence does not always work as expected [15]. Since a correlation between the anxiety of children and their parents has been demonstrated during the preoperative period [16], and the dramatic changes in psychology and behaviour in the development of children, it is suggested that tailored preoperative sedation approaches should be established on individual characteristics of children and their parents [17, 18]. Evidence showed that an empathic patient-centred attitude in preoperative period can significantly reduce patient anxiety [19, 20], and patient- or family-centred care could be a critical approach to improve the quality of health care [21]. This may suggest that a patient- or family-centred approach is an ideal way to manage paediatric preoperative anxiety.

## Limitations

Although all tertiary maternity and children’s hospitals registered with the National Health Commission of the People’s Republic of China were invited to participate in our study, it is still difficult to ensure that these results comprehensively describe the current status of paediatric preoperative sedation services. Because only one anaesthesiologist from each hospital was invited to participate in the survey, these responses may not accurately represent the practice of all anaesthesiologists within the institution. In addition, experiences reported by tertiary hospitals may not be representative of lower-level hospitals because of variability in the workforce, facilities, and knowledge, which may also have an impact on the results.

## Conclusions

We concluded that paediatric preoperative sedation services vary widely in tertiary maternity and children’s hospitals in China. There are still challenges and areas for improvements, more work is needed to establish an evidence-based standard operation procedure to improve the quality of paediatric preoperative sedation services. Although there are still some limitations in this study, the hospitals included in our study did represent the tertiary centres providing paediatric service in different provinces across China, we hope this study to be a starting point to contribute to the future establishment of a national consensus procedure to achieve a national guideline regarding patient comfort and safety.

## Data Availability

The datasets used and analysed during the current study are available from the corresponding author upon request.

## Abbreviations

ASA: American Society of Anesthesiologists
